# Neurobehavioral Responses and Toxic Brain Reactions of Juvenile Rats Exposed to Iprodione and Chlorpyrifos, Alone and in a Mixture

**DOI:** 10.3390/toxics11050431

**Published:** 2023-05-05

**Authors:** Yasmina M. Abd-Elhakim, Nabela I. El Sharkawy, Heba S. A. Gharib, Mona A. Hassan, Mohamed M. M. Metwally, Khlood M. Elbohi, Bayan A. Hassan, Amany Tharwat Mohammed

**Affiliations:** 1 Department of Forensic Medicine and Toxicology, Faculty of Veterinary Medicine, Zagazig University, Zagazig 44519, Egypt; monaabdelhady91@yahoo.com (M.A.H.); kmelbohy@zu.edu.eg (K.M.E.); amany_tharwat@yahoo.com (A.T.M.); 2 Department of Behaviour and Management of Animal, Poultry, and Aquatics, Faculty of Veterinary Medicine, Zagazig University, Zagazig 44519, Egypt; dr_heba_vet@yahoo.com; 3 Department of Pathology, Faculty of Veterinary Medicine, Zagazig University, Zagazig 44519, Egypt; mmetwally@zu.edu.eg; 4 Pharmacology Department, Faculty of Pharmacy, Future University, Cairo 11835, Egypt; bayan.saffaf@fue.edu.eg

**Keywords:** iprodione, chlorpyrifos, brain, anxiety, depression, acetylcholinesterase, oxidative stress

## Abstract

Herein, male juvenile rats (23th postnatal days (PND)) were exposed to chlorpyrifos (CPS) (7.5 mg/kg b.wt) and/or iprodione (IPD) (200 mg IPD /kg b.wt) until the onset of puberty (60th day PND). Our results demonstrated that IPD and/or CPS exposure considerably reduced locomotion and exploration. However, CPS single exposure induced anxiolytic effects. Yet, neither IPD nor IPD + CPS exposure significantly affected the anxiety index. Of note, IPD and/or CPS-exposed rats showed reduced swimming time. Moreover, IPD induced significant depression. Nonetheless, the CPS- and IPD + CPS-exposed rats showed reduced depression. The individual or concurrent IPD and CPS exposure significantly reduced TAC, NE, and AChE but increased MDA with the maximum alteration at the co-exposure. Moreover, many notable structural encephalopathic alterations were detected in IPD and/or CPS-exposed rat brain tissues. The IPD + CPS co-exposed rats revealed significantly more severe lesions with higher frequencies than the IPD or CPS-exposed ones. Conclusively, IPD exposure induced evident neurobehavioral alterations and toxic reactions in the brain tissues. IPD and CPS have different neurobehavioral effects, particularly regarding depression and anxiety. Hence, co-exposure to IPD and CPS resulted in fewer neurobehavioral aberrations relative to each exposure. Nevertheless, their simultaneous exposure resulted in more brain biochemistry and histological architecture disturbances.

## 1. Introduction

Pesticide production and use has risen to satisfy the increasing worldwide demand for food [1]. There has been a rise in the use of pesticides, herbicides, and fungicides to boost crop yields [2,3]. Consequently, various types of pesticides are continuously released into the environment, which humans and animals are inevitably exposed to and affected by [4,5]. Similarly, insecticides are routinely combined with fungicides in agricultural fields for simultaneous application [6]. Organophosphorus insecticides (OPIs) are the most widely utilized agrochemicals worldwide [7]. Chlorpyrifos (CPS) has become one of the most commonly used OPIs in agriculture, industry, and gardening globally in recent decades [8,9]. According to 2016 European Union data, CPS is among Europe’s top 15 pesticides identified in food products [10]. CPS accumulates, exceeding the permissible residue limits of 0.2, 0.3, 0.5, and 0.5 mg/kg for peaches, oranges, grapes, and tomatoes, respectively [11]. Moreover, CPS residues are usually detected in citrus fruits, apples, bananas, and pears [9]. Many adverse effects were caused by CPS exposure, such as neurotoxicity [12], nephrotoxicity [13], developmental toxicity [14], reprotoxicity [15,16], endocrine disruption [17], and hepatotoxicity [18,19].

Neurobehavioral problems and CPS exposure in humans are major environmental health issues, particularly in children, who are highly susceptible to environmental dangers [20]. Children’s brains are particularly vulnerable to the effects of pesticides because of their high rate of circuitry and architecture development [21]. In rats, the ability to metabolize CPS appears to be age-dependent, with pups having poorer detoxification due to their lower metabolic maturity [22]. Inhibition of the acetylcholinesterase enzyme (AChE) in the peripheral and central nervous system is the principal mechanism by which CPS causes neurotoxicity [23]. Additionally, previous research verified that CPS causes oxidative stress in the growing rat brain, producing neuronal damage by increasing the generation of reactive oxygen species (ROS), lipid peroxidation, and DNA damage in the CNS [24,25]. Moreover, it has been shown that oxidative stress is the principal mechanism of CPS toxicity in chronic or subchronic exposure [23,26]. Furthermore, CPS reduces nerve cell replication and neurite formation and intervenes in signaling cascades and transcriptional events in neural cell differentiation [25].

Using fungicides is essential in cultivating fruits and vegetables, thus ubiquitously distributed in the environment [27]. Iprodione (IPD) is a popular dicarboximide fungicide used in producing tomatoes, green beans, grapes, and strawberries [28]. Although IPD is less toxic than organophosphate and organochlorine fungicides, it threatens the environment due to its widespread application and long half-life. In this regard, research on IPD residues in a wide range of crops, such as tomato [29], peppers [30], cabbage [31], green tobacco leaves [32], green beans [33], strawberries [34], and grapes [35], has shown that IPD has a substantially longer half-life than other fungicides. IPD residues were also identified in garlic, grapes, and certain small tropical crops (Indian jujube, wax apple, and starfruit) where its use was not registered [36,37,38]. Additionally, there is a greater risk of IPD accumulating at levels higher than those authorized by EU-established MRLs in greenhouse crops [31]. Indeed, among 23 pesticides tested for residual detection in fruit products, IPD was shown to be the most prevalent [39]. In some experimental models, IPD showed endocrine disruption [40,41], cardiotoxicity [42], and reprotoxicity [15,16]. Nonetheless, the evidence on IPD toxicity is still inadequate and does not allow for risk assessments [43]. For many years, it was thought that neurotoxicity studies on IPD were not required since IPD is neither an organophosphate nor structurally related to compounds known to induce neurotoxicity [44]. However, in a recent study on zebrafish, IPD seriously damages the brain [45]. Yet, research has to identify the probable IPD-induced neurotoxic consequences in rats.

It is hypothesized that when these pesticides are applied simultaneously, they may have an additive or synergistic effect. Despite this, the results of combined exposure to IPD and CPS on the neurological system have not yet been explored. Consequently, this study assessed the outcome of the individual or combined exposure to IPD and CPS on locomotor activity, anxiety, depression, muscular activity, brain architecture, neurotransmitter levels, and biomarkers of oxidative stress and lipid peroxidation.

## 2. Materials and Methods

### 2.1. Tested Compounds

The commercially available form of IPD (Ippon, 50% suspension concentrate, SC comprises 50% IPD and 50% other ingredients) was purchased from Arysta Life Science- Egypt Ltd., Cairo, Egypt. CPS in the form of Pestban (48% CPS, 32% solvent, and 20% surface-active compounds) was purchased commercially from the National Corporation for Agrochemicals and Investment-AGROCHEM (Alexandria, Egypt). Stock IPD and CPS solutions were made using corn oil. All other chemicals were gained from Sigma Aldrich Co. (St. Louis, MO, USA).

### 2.2. Animals and Experimental Design

Forty male immature Sprague Dawley rats (23rd postnatal day (PND); 65 ± 0.20 g average initial weight) were obtained from the Animal House of the Faculty of Veterinary Medicine, Zagazig University, Egypt. The rats were fed ad libitum a standard pellet diet and given free access to water. Four groups of ten rats each were enrolled in this experiment. The control group was orally gavaged 1 mL corn oil/rat. The IPD group was orally gavaged 200 mg IPD/kg b.wt equal to 1/20 LD_50_ [40]. The CPS group was orally gavaged 7.45 mg CPS/kg b.wt equal to 1/20 LD_50_ [46]. The co-exposed group was orally given both IPD and CPS at the same aforementioned doses (1/20 LD_50_) and route. All treatments were given until the onset of puberty at the 60th day PND [47]. Weekly body weights of all rats were recorded and used to calibrate dosage volumes.

### 2.3. Neurobehavioral Assessments

Neurobehavioral tests, such as open field test (OFT), forced swimming test (FST), and elevated plus maze (EPM) tests, were performed on the last 2 days of the experiment in the same testing room to investigate the behavioral changes of rats following administration with CPS and/or IPD. Before behavioral testing, the animals were transferred to the testing room 20 min prior to the testing to accommodate the surrounding condition. The animal group’s identity was unknown to the observer. The EPM test was performed between 1.00 and 3.00 p.m., and on the next day, rats were submitted to the OFT in the morning (between 8.00 and 10.00 a.m.) and to the FST between 1.00 and 3.00 p.m.

#### 2.3.1. Open Field Test

The OFT is usually used to judge rodents’ locomotor activity and emotional responses. For open field observation, each rat was retained in the arena center of the pen field apparatus [48] and permitted to discover for 5 min freely. These sessions were video recorded and evaluated by an investigator blind to experimental groups. The following behavioral indices were recorded: the total number of square crossed (movement of all 4 paws in the animal across a square and entrance in another), rearing frequency (counts of how many times the animal stood on its hind legs), grooming activities frequency, freezing duration (phase in seconds that the rat was motionless) and stretched attenuated posture (SAP) frequency (number of elongations of the rat’s head and shoulder after which the original position was reclaimed). After each animal test session, the field was cleaned with 5% ethanol.

#### 2.3.2. Elevated plus Maze

Elevated plus maze (EPM) was commonly used to evaluate anxiety-like behavior in rodents [49]. In line with the protocol of Sánchez-Amate, et al. [50], The apparatus consisted of two opposing open arms and two closed arms (each 10 × 50 cm) which extended from a common central square plot (10 × 10 cm). The entire apparatus was high, 50 cm from the floor. Each rat was kept in the center area of the EPM facing one of the open arms and was permitted to discover the EPM apparatus for 5 min under conditions of no noise and minimal movement. The maze was cleaned with 10% ethanol before introducing each animal to avoid odors and/or residues left by rats tested earlier. A video camera recorded the following behavior: the number of closed and open arm entries and the duration of which rats remained in the closed and open arms. The anxiety index (Y) was determined using the following formula:

(1) $$Y=1-[(\frac{a}{b}+\frac{C}{d})/2]$$

**where *a* =** time the animal stayed in the open arms (seconds), *b* = test duration (300 s), *c* = input frequency in the open arms, and *d* = total number of entries.

#### 2.3.3. Forced Swimming Test (FST)

FST was performed based on the procedures defined by Porsolt, et al. [51]. The test was conducted in two stages, the pre-test and the test. Each animal was kept in a glass cylinder (45 cm height and 30 cm diameter) containing 25 cm of water (25 °C ± 2) for 15 min (duration of pre-test session). After 24 h of the pre-test, the rats were exposed to the test sessions, which was 4 min for each rat. The predominant indicative behavior of depression, including swimming and immobility (the rat was floating in the water without struggling and only making movement necessary to keep its nose above water) were recorded. The water of the aquarium was exchanged after each animal.

### 2.4. Sampling

At the end of the experiment, the rats were anesthetized by intraperitoneal injections of 100 mg/kg b.wt pentobarbital sodium and euthanized by decapitation. Following saline washing, the brain tissue samples were divided into two sets. The first was homogenized, centrifuged at 664× *g* at 4 °C for 15 min, and the resultant supernatant was used for biochemical analysis. The second brain half was kept in 10% neutral buffered formalin for histopathological assessments.

### 2.5. Biochemical Assessment of Brain Tissue Homogenate

#### 2.5.1. Assessment of Brain AChE and Nor-Epinephrine (NE) Levels

In the brain tissue homogenate, AChE has been evaluated using ELISA Kit (CUSABIO, Houston, TX, USA) (Catalog no. CSB-E11304r; sensitivity: 1.95 pg/mL, detection range: 7.8–500 pg/mL). Additionally, NE brain content was estimated using Rat (NE) ELISA Kit (MyBioSource, San Diego, CA, USA, Catalog no. Cat No.MBS269993, detection range: 31.2–2000 pg/mL).

#### 2.5.2. Measurement of Oxidative Stress and Apoptosis Biomarkers in Brain Homogenates

Total antioxidant capacity (TAC) was assessed by the colorimetric technique, by the sample antioxidant reaction with a defined hydrogen peroxide (H_2_O_2_) amount [52]. Then, the residual H_2_O_2_ is quantified through the sample’s antioxidant reaction involving the transformation of 3,5,dichloro-2-hydroxybenzensulphonate to a colored product. Malondialdehyde (MDA) was measured by the reaction with thiobarbituric acid in an acidic medium for 30 min at 95 °C to produce a pink reactive product determined at 534 nm [53].

### 2.6. Histopathological Evaluation

After euthanasia, standard necropsy procedures were performed, and tissue specimens from the right cerebral hemisphere were collected from all rats following the guidelines of Ruehl-Fehlert, et al. [54]. The samples were fixed in 10% phosphate-buffered formalin for 72 h, carefully washed in distilled water, dehydrated by passing through a series of graded ethyl alcohol (70%, 80%, 90%, and 100%), and cleared in HistoChoice^®^ clearing agent (Scientific Laboratory Supplies Ltd., Nottingham, UK). The cleared specimens were impregnated and embedded in a paraffin–beeswax mixture (10% beeswax and 90% paraffin wax), sectioned 5 µm thick, and stained with hematoxylin and eosin dyes following the staining protocol described by Suvarna, et al. [55]. The stained tissue sections were inspected microscopically, and ten nonoverlapped randomly chosen microscopic fields (High power field (HPF), 40×) of fixed sizes (220 × 280 µm) per rat (100 images per group) were snapshotted by AmScope digital camera (United Scope LLC, Irvine, CA, USA) attached to Nikon light microscope (Nikon Instruments Inc., New York, NY, USA). Next, a multiparametric numerical lesion scoring was established where the images were analyzed to measure the CPS and/or IPD-induced histological encephalopathic changes. The quantified changes were (1) the percentages of neurons manifested chromatolysis, perineuronal vacuolation, and neuronal necrosis regarding the total numbers of neurons per image, (2) the percentages of the area fractions of the intracerebral blood vessels, meningeal blood vessels, choroidal plexus blood vessels, intracerebral hemorrhages, meningeal hemorrhages, and neuropil microcavitations regarding the total areas of the images using the open source ImageJ software 1.8.0 [56], and (3) the frequencies of glial clustering, gliosis, satellitosis, neuronophagia, endothelial hypertrophy, and cerebral and meningeal inflammatory cell infiltration regarding the total number of images/group by the following formula: Lesion FQ (%) = N_lesion_ × N_total_^−1^ × 100. N_lesion_ = the number of images showing the lesion. N_total_ = the total number of images/group (100 images). The results were expressed as percentages (means ± SE).

### 2.7. Data Analysis

The data’s normality and variance homogeneity were examined using Kolmogorov–Smirnov and Levene’s tests, respectively. When normality assumptions were met, data were analyzed using IBM SPSS Statistics version 21 (IBM; Armonk, New York, NY, USA) by one-way analysis of variance (ANOVA) to statistically define the variation between groups, followed by Tukey’s multiple range post hoc test for pairwise comparisons. The data have been displayed as means ± SE. At *p* < 0.05, mean differences were considered significant. Moreover, GraphPad Prism version 8 (GraphPad Software, San Diego, CA, USA) was used for data presentation [57]. The principal component analysis was applied to the replicates of all analyses by the Granato, et al. [58] method.

## 3. Results

### 3.1. Effects on Locomotors Activity and Anxiety in OFT

**Figure 1:**
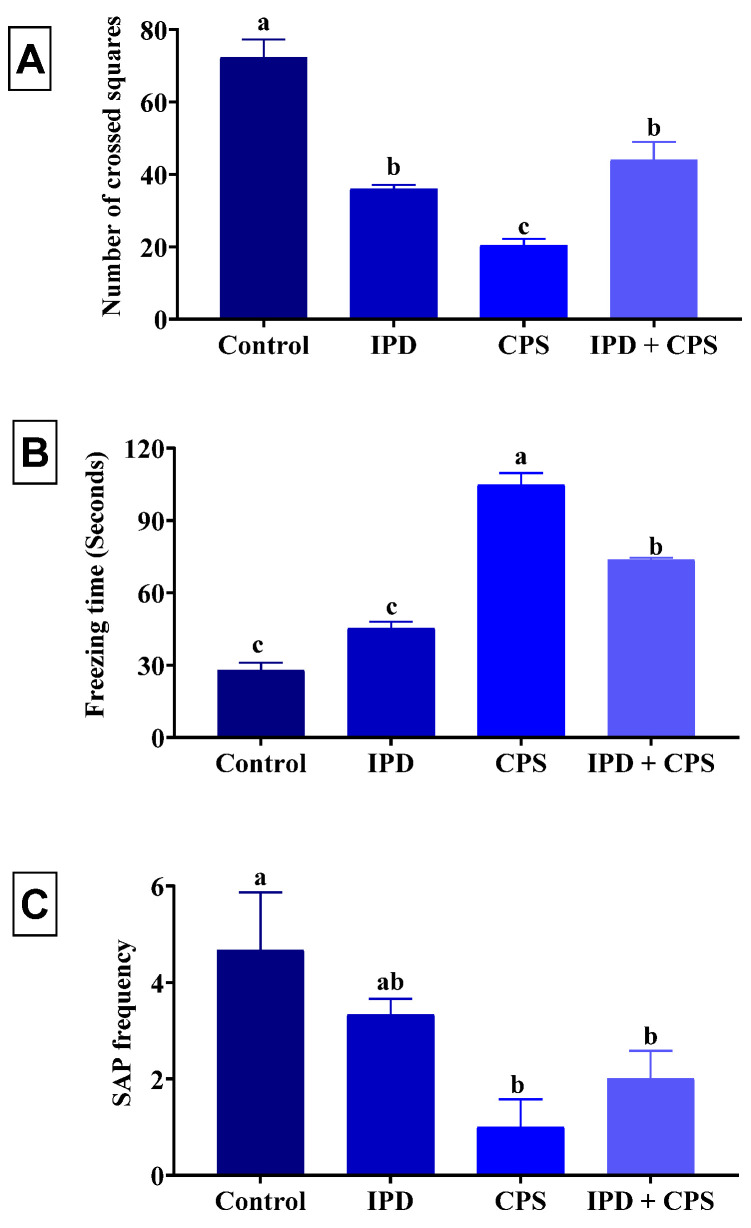
Effect of iprodione (IPD) and/or chlorpyrifos (CPS) exposure on the number of crossed square (**A**), freezing time (**B**), and stretched attenuated posture (SAP) (**C**) frequency of juvenile Sprague Dawely male rats in the open field test. Data expressed as mean ± SE, n = 10 for each group. Each bar carrying different letters (a–c) was significantly different at *p* < 0.05.

In the OFT, the IPD, CPS, and IPD + CPS-exposed rats showed a reduced locomotor activity represented a significantly (*p* < 0.001) reduced number of crossed squares by 50%, 72%, and 39%, receptively, compared to the non-exposed ones (Figure 1A). Moreover, a significant increase in the freezing time by 62%, 273%, and 163% was recorded in the IPD, CPS, and IPD + CPS-exposed rats, receptively, compared to control ones (Figure 1B). The SAP frequency was significantly (*p* = 0.04) decreased in the rats exposed to CPS and IPD + CPS by 79% and 57%, receptively, compared to the control ones (Figure 1C).

**Figure 2:**
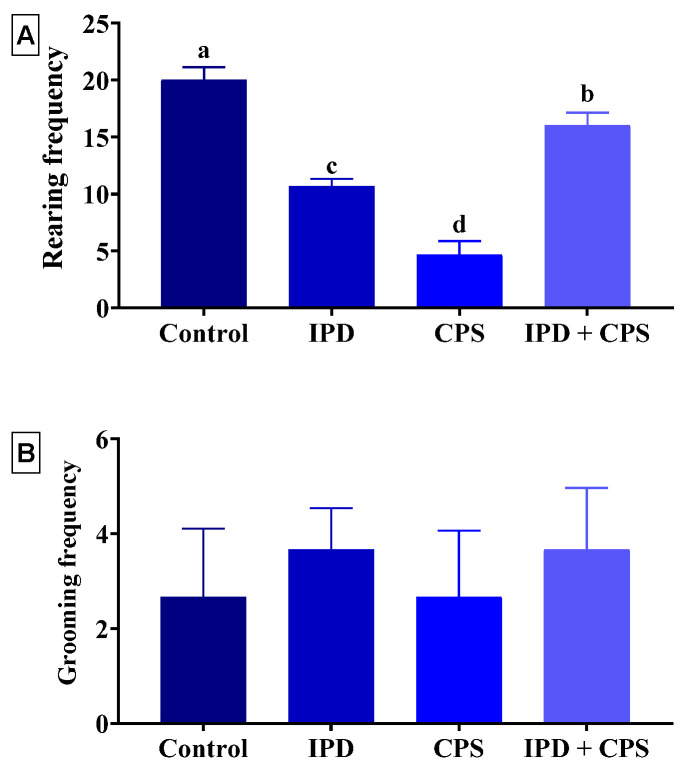
Effect of iprodione (IPD) and/or chlorpyrifos (CPS) exposure on the rearing frequency (**A**) and grooming frequency (**B**) of juvenile Sprague Dawely male rats in the open field test. Data expressed as mean ± SE, n = 10 for each group. Each bar carrying different letters (a–d) was significantly different at *p* < 0.05.

As displayed in Figure 2A, the rearing frequency was significantly (*p* < 0.001) decreased in the IPD, CPS, and IPD + CPS-exposed rats by 47%, 77%, and 20%, respectively, compared to the control ones. Nevertheless, no significant alteration in the grooming frequency was recorded among rats in different experimental groups (Figure 2B).

### 3.2. Effects on Depression in the FST

**Figure 3:**
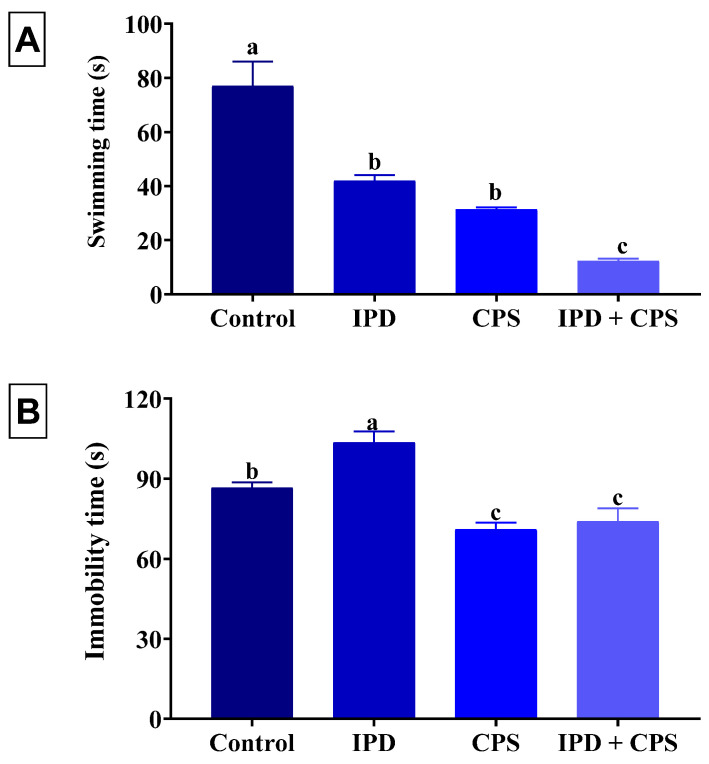
Effect of iprodione (IPD) and/or chlorpyrifos (CPS) exposure on the swimming time (**A**) and immobility time (**B**) of juvenile Sprague Dawely male rats in the forced swimming test. Data expressed as mean ± SE, n = 10 for each group. Each bar carrying different letters (a–c) was significantly different at *p* < 0.05.

As shown in Figure 3, a significant (*p* < 0.001) reduction in the swimming time of 45%, 59%, and 84% was recorded in the IPD, respectively, in CPS, and IPD + CPS-exposed rats, receptively, compared to the non-exposed ones. Of note, the IPD and CPS co-exposed rats displayed a significantly lesser swimming time than those exposed to each separately. On the other hand, the rats exposed to IPD showed a significant (*p* = 0.001) increased immobility time by 20% than the control group. However, a significant (*p* = 0.001) reduction in the immobility time was recorded in the FST in juvenile rats independently exposed to CPS and those co-exposed to IPD and CPS by 18% and 15%, respectively, compared to the control group.

### 3.3. Effects on Anxiety in EPM

**Figure 4:**
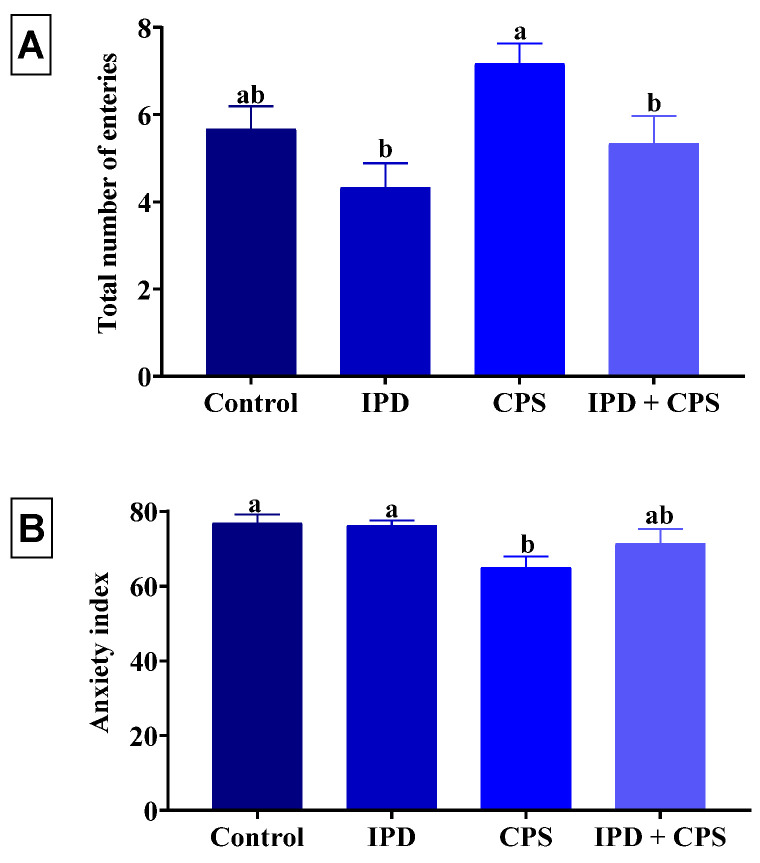
Effect of iprodione (IPD) and/or chlorpyrifos (CPS) exposure on total number of entries in the open and closed arm (**A**) and anxiety index (**B**) of juvenile Sprague Dawely male rats in the elevated plus maze test. Data expressed as mean ± SE, n = 10 for each group. Each bar carrying different letters (a and b) was significantly different at *p* < 0.05.

Neither the individual exposure to IPD nor the co-exposure to IPD and CPS had altered the total number of open and close arm entries and the anxiety index compared to the control group (Figure 4). Nonetheless, the single exposure to CPS significantly (*p* = 0.02) decreased the anxiety index by 16% compared to the control group without significant change in the total close and open arm entries.

### 3.4. Effects of Brain AChE and NE Neurotransmitter

**Figure 5:**
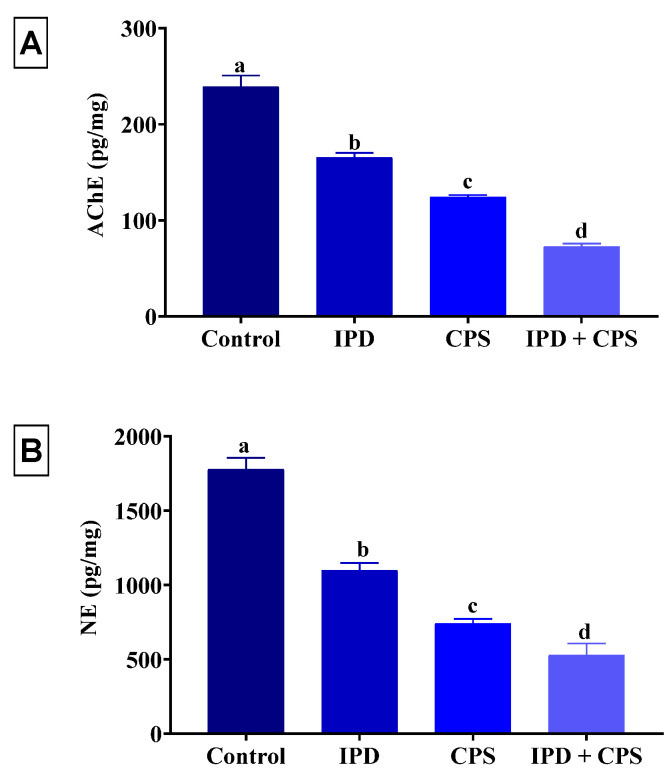
Effect of iprodione (IPD) and/or chlorpyrifos (CPS) exposure on acetylcholinesterase (AChE) activity (**A**) and norepinephrine (NE) (**B**) of the brain tissue of juvenile Sprague Dawely male rats. Data expressed as mean ± SE, n = 10 for each group. Each bar carrying different letters (a–d) was significantly different at *p* < 0.05.

As demonstrated in Figure 5, a significant (*p* < 0.001) decrease in the brain content of AChE and NE was recorded in the IPD (31% and 38%, respectively) or CPS (48% and 58%, respectively) exposed rats compared to the control group. Notably, rats exposed to both IPD and CPS had a significantly (*p* < 0.001) lower AChE and NE brain content than those exposed to either alone and their values were 70% lower than the control rats.

### 3.5. Effects on Brain Oxidative Status

**Figure 6:**
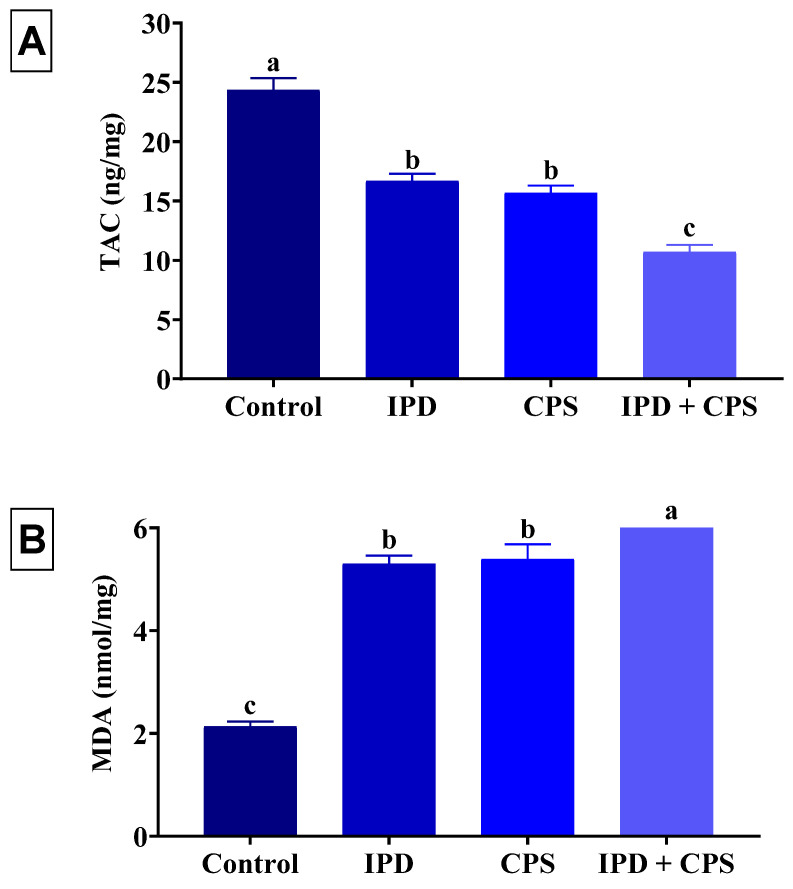
Effect of iprodione (IPD) and/or chlorpyrifos (CPS) exposure on total antioxidant capacity (TAC) (**A**) and malondialdehyde (MDA) (**B**) of the brain tissue of juvenile Sprague Dawely male rats. Data expressed as mean ± SE, n = 10 for each group. Each bar carrying different letters (a–c) was significantly different at *p* < 0.05.

The effects of oral exposure to IPD and/or CPS for 37 days on the oxidative status and lipid peroxidation level of the brain tissues of juvenile rats are displayed in Figure 6. The IPD or CPS-exposed rats exhibited a significant (*p* < 0.001) depletion of TAC by 31% and 36%, respectively, compared to the control ones. However, the brain MDA content of IPD or CPS-exposed rats was significantly (*p* < 0.001) increased by 149% and 153%, respectively, compared to the control rats. On the other hand, the IPD and CPS co-exposed rats had a significantly (*p* < 0.001) lower TAC (56%) and higher (194%) MDA than the control group. Of note, the IPD and CPS co-exposed rats showed a significantly lower TAC but higher MDA brain content than those exposed to each individually.

### 3.6. Histopathological Findings

**Figure 7:**
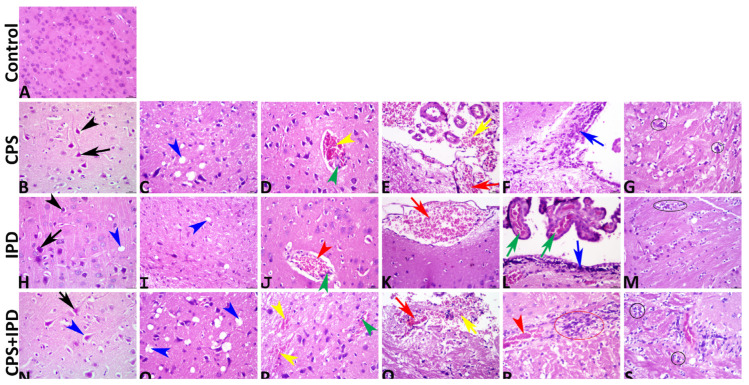
Representative photomicrographs of H&E-stained brain tissue sections showing normal histology in the control group (**A**). The chlorpyrifos (CPS) (**B**–**G**), iprodione (IPD) (**H**–**M**), and CPS + IPD (**N**–**S**) groups show numerous encephalopathic histological alterations varying from severe in the CPS to less severe in the IPD to much more severe in the CPS + IPD group. The symbols on the images denote the following: black arrowhead, perineuronal vacuolation; black arrow, necrotic neuron; blue arrowhead, neuropil microcavitation; blue arrow, subependymal mononuclear cell infiltration; yellow arrowhead, intracerebral hemorrhage; yellow arrow, meningeal hemorrhage; green arrowhead, endothelial hypertrophy; green arrow, choroid plexus congestion; red arrowhead, intracerebral congestion; red arrow, meningeal congestion; red ellipse, intracerebral mononuclear cell infiltration; black ellipse, gliosis.

The microscopic examination and the data obtained from the image analysis declared that no histological alterations were found in the control group, which exhibited normal cerebral and meningeal architectures (Figure 7A). Exposure to CPS in a dose of 7.45 mg/kg b.wt for 37 successive days induced a vast array of notable structural encephalopathic alterations. Primarily, these alterations were degenerative, necrotic, and circulatory, while the inflammatory response was barely detectable. Most tissue sections of this group exhibited necrotic neurons with shrunken hypereosinophilic cytoplasm and pyknotic deeply basophilic nuclei with perineuronal vacuolation (Figure 7B), obvious neuropil microcavitations (Figure 7C), intracerebral and meningeal congestions and hemorrhages with endothelial hypertrophy (Figure 7D,E), and gliosis (Figure 7G). Few tissue sections showed mononuclear cell infiltration, particularly in the subependymal spaces (Figure 7F). Similar to CPS, exposure to IPD in a dose of 200 mg/kg b.wt for 37 successive days induced numerous degenerative, necrotic, and circulatory encephalopathic alterations, yet the IPD-induced lesions were significantly lower in severity and markedly lesser in frequency in comparison to the CPS-induced lesions. Most tissue sections of this group manifested few neurons possessing pyknotic nuclei and hypereosinophilic cytoplasm with tiny perineuronal vacuolations (Figure 7H), infrequent neuropil microcavitations (Figure 7I), intracerebral congestions with nearly absent intracerebral hemorrhages (Figure 7J), meningeal congestion, sometimes associated with minute meningeal hemorrhages (Figure 7K), and glial clustering (Figure 7L). Subependymal mononuclear cell infiltration accompanied by choroidal congestions was seen in a few tissue sections (Figure 7M). Co-exposure to CPS and IPD exacerbated the structural encephalopathic effects of each other, as the brains of the CPS + IPD-treated animals revealed significantly more severe lesions with higher frequencies than either the CPS or the IPD-treated animals. All tissue sections of this group showed pronounced histopathological alterations, particularly the necrotic and circulatory changes. Numerous necrotic neurons with perineuronal vacuolation (Figure 7N), dispersed neuropil microcavitation (Figure 7O), intracerebral congestions, and hemorrhages (Figure 7P), meningeal congestions, and hemorrhages (Figure 7Q), and intracerebral mononuclear cell infiltrations (Figure 7R), and gliosis (Figure 7S) had consistent findings. Precise summaries for all lesions with their quantification among all groups are tabulated in Table 1.

### 3.7. Correlation Analysis of the Estimated Parameters

**Figure 8:**
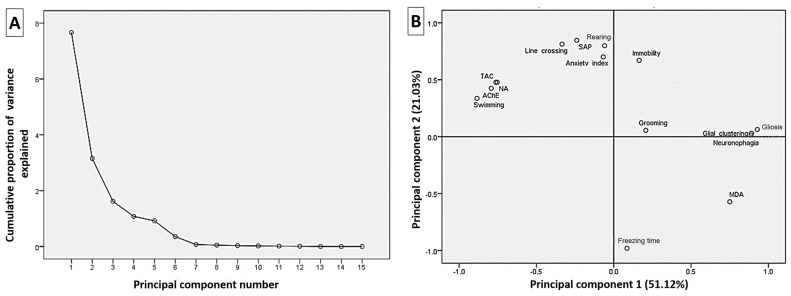
The principal component analysis plot shows the estimated variables’ relationships. (**A**) Cumulative proportion of variance as a function of the number of principal components (PC). (**B**) All biochemical and neurobehavioral indicators are plotted as a function of PC1 and PC2, which account for 51.12% and 21.03% of the variance, respectively. SAP: stretched attenuated posture; AChE: acetylcholinesterase activity; NE: norepinephrine; TAC: total antioxidant capacity; MDA: malondialdehyde.

Principal components analysis was used to examine the correlation between the analyzed variables and 15 components were attained (Figure 8A). As shown in Figure 8B, the loading plot of the first two components (51.12% and 21.03%) represented approximately 72.15% of the overall variation in the experimental data. The variables (<90°) apart on the loading plot are positively correlated and strongly correlated. Accordingly, TAC, AChE, NE, swimming time, line crossing, anxiety index, SAP, and rearing frequency variables are grouped together and highly correlated with the first component. Moreover, the MDA was correlated with the second component and was highly negatively correlated with the first component variables.

## 4. Discussion

Adolescence is arguably as important to brain development as the prenatal and postnatal periods [59]. Environmental factors and other neurotoxic stressors can potentially affect the adolescent brain [60]. Hence, the current study evaluated the effect of single or combined exposure to IPD or CPS on neurobehavioral performance and brain biochemistry in adolescent rats.

The OFT allows for assessing xenobiotic-related effects on many aspects of animal behavior, such as locomotor activity, exploratory behavior, and anxiety. Initially, the number of crossed squares, the freezing duration, and the rearing frequency are commonly used as measures of locomotion and exploration [61]. In the current experiment, IPD and/or CPS-exposed rats showed notable reduced locomotion and exploration in the OFT. In this regard, Kudavidanage, et al. [62] confirmed that oral CPS dosing impaired coordination and decreased muscle strength in rats. On the other hand, AChE inhibition has been reported to disrupt motor function [63]. Thus, the recorded reduction of AChE in the brain tissues of IPD and/or exposed group could be responsible for the reduced locomotion.

Anxiety is one of the behaviors most affected by pesticide exposure [64,65]. The current study uses several behavioral tests to assess anxiety, including grooming and SAP in OFT and anxiety index in EPM. The importance of grooming behavior as an anxiety indicator has been documented [66]. Moreover, SAP can be used as a reliable indicator of anxiety as it is reduced by anxiolytic medicines [67]. The CPS-exposed rats showed an anxiolytic-like behavior as represented by reduced SAP in the OPT and anxiety index in EPM. The decreased locomotor activity in OFT in CPS can be related to anxiolytic-like behavior. Likewise, the EPM findings in the study of Chen, et al. [68] verified that repeated CPS exposure resulted in an anxiolytic-like effect at relatively high doses but with a short exposure time (7 days). There was a link between anxiety pharmacology and cholinesterase inhibitor behavioral toxicity [50]. Degroot and Treit [69] indicated that augmented brain ACh levels decrease anxiety, which agrees with our outcomes of reducing anxiety degree with the decreased AChE. Likewise, Schulz, et al. [70] reported that when rats were repeatedly exposed to another OPI, parathion-methyl, in the EPM test, their anxiety level decreased. On the contrary, the anxiogenic effects of acute exposure to large doses of CPS were observed 48 h after injection in rats [50]. Nonetheless, mounting evidence suggested that CPS causes neurotoxicity at low doses by mechanisms distinct from those seen at higher doses [71,72]. On the other hand, despite the reduction of locomotor activity and AChE and NE in the brain of IPD and IPD + CPS-exposed rats, no significant changes were recorded in the SAP and grooming in the OPT or the anxiety index in EPM in these groups. This could be related to the several factors controlling anxiety disorders, including other neurotransmitters, such as γ-aminobutyric acid [73], serotonin, and dopamine [74]. Hence, investigating the effect of IPD exposure on other neurotransmitters is highly needed in further studies to unravel the possible underlying mechanisms associated with its neurobehavioral effects. On the other hand, the disappearance of the CPS-anxiolytic activity in the IPD + CPS-co-exposed rats could be linked to the exaggeration of the oxidative stress in the brain tissues as revealed biochemically in the co-exposed group than the other groups. In this regard, several studies confirmed the higher anxiety associated with oxidative stress [75,76,77] which could mask the CPS-anxiolytic activity in our case.

Herein, the behavioral responses associated with depression were assessed by FST. IPD and/or CPS-exposed rats showed notably reduced swimming time. However, the immobility time, considered the primary indicator of depression, has significantly increased in the IPD and IPD + CPS groups. Initially, the IPD-exposed group showed an apparent depressant effect regarding a significant increase in the immobility time. The IPD-induced depression could be related to the decreased AChE activity. Prolonged AChE inhibition in the brain may raise ACh levels at cholinergic synapses. As a result, elevated ACh signaling can result in depression-related symptoms [78,79]. Yet, the immobility time was markedly reduced in the CPS and IPD + CPS groups despite the reduction of AChE. Thus, we propose that the decrease in swimming time could be related to the motor impairment in the CPS group rather than indicative of depression. Comparably, with repeated subcutaneous injections of 160 mg/kg, CPS considerably decreased immobility time, displaying an antidepressant-like activity [68]. Oppositely, Chen, et al. [20] found that injecting CPS 10 or 20 mg/kg subcutaneously into adolescent male rats between 27–36th PND resulted in depressive-like behavior. Hence, the CPS-associated neurobehavioral performance dramatically varies with the dose, duration, or route of exposure. Of note, the discrepancies in the effect of IPD, CPS, and IPD + CPS exposure on depression, despite their similar inhibitory effect on AChE, could be related to their different modulatory effect on other neurotransmitters. In this regard, several studies demonstrated the complexity of the interplay of different neurotransmitters, such as serotonin and dopamine [80,81] in the occurrence of depression disorder. Thus, this point needs further investigation.

In the current study, a prominent inhibition of AChE was apparent in the brain tissues of IPD and/or CPS-exposed rats. Several investigations have shown that CPS’s toxic effects originate from its ability to inhibit AChE activity in the brain and the rest of the nervous system [68,82]. Young animals are more vulnerable to the harmful effects of cholinesterase-inhibiting pesticides, and young rats (PND 17) had identical behavioral abnormalities and AChE inhibition at a five-fold lower dose than adult rats [83]. Gallegos, et al. [84] proposed that many mechanisms, such as changes in enzyme turnover, gene and protein expression, or the death of AChE-expressing cells, may contribute to the CPS-induced decrease in AChE activity. Several earlier reports have confirmed that long-term CPS exposure affects the cholinergic system and a wide range of neurotransmitter systems. Studies showed that CPS might also interfere with the metabolism of monoamine transmitters, such as dopamine and epinephrine [85,86] and serotonin [87]. Herein, a significant depletion of NE was apparent in IPD and/or CPS-exposed rats. The reduced NE brain level could be responsible for the CPS-induced anxiolytic activity.

In the current study, considerable oxidative stress and peroxidative lipid damage of the brain tissues was evident in the rats exposed to IPD or CPS, with the maximum damage in the IPD or CPS co-exposed rats. Comparably, IPD has been reported to deplete the antioxidant content of the heart in zebrafish [42] and testes in rats [16]. Moreover, IPD induced ROS production in numerous cell lines in vitro [1,88]. Additionally, several studies have found that CPS and IPD exposure increases ROS generation [43,89]. Consequently, the reported histopathological perturbations in the brain tissues of the IPD-exposed rats, including meningeal congestion, meningeal hemorrhages, glial clustering, subependymal mononuclear cell infiltration, and choroidal congestions, could be closely linked to its oxidative and lipid peroxidative-inducing effect. In this regard, in the recent study of Lai, et al. [45], the brain of the zebrafish exposed to IPD was significantly damaged.

Of note, IPD and CPS significantly resulted in opposing neurobehavioral alterations or have different neurobehavioral effects. Nevertheless, a synergistic consequence was noted with IPD and CPS joint exposure in biochemical parameters, including TAC, MDA, AChE, and NE. CPS and other pesticides, such as permethrin [90] and imidacloprid [91], have similar synergistic interactions. The coexistence of oxidative stress and low AChE activity in brain tissue may have contributed to the exacerbated pathological changes described in the brain tissues of adolescent rats co-exposed to IPD and CPS.

## 5. Conclusions

The current findings revealed for the first time the harmful impacts of exposure to IPD on the neurobehavioral response and brain biochemistry and architecture of juvenile rats. Moreover, the IPD and CPS exposure induced a different effect on neurobehavioral performance, consequently reducing the neurobehavioral alteration at their co-exposure. Nevertheless, our findings provide a unique perspective that increased ROS production after IPD and CPS co-exposure may exaggerate brain injury. Such findings explain the importance of exploring the outcomes of exposure to pesticide mixtures. However, further investigation into these pesticides’ interactions is needed.

## Figures and Tables

**Table 1:** Effect of chlorpyrifos (CPS) and/or iprodione (IPD) exposure on the brain histology of juvenile Sprague Dawely male rats.

	Lesion	Control	IPD	CPS	IPD + CPS
Percentage of neurons exhibited the lesion to the total number of neurons per images per group	Chromatolysis	0.00 ^b^ ± 0.00	3.00 ^a^ ± 1.53	5.00 ^a^ ± 2.24	11.00 ^a^ ± 3.14
	Perineuronal vacuolation	0.00 ^c^ ± 0.00	6.00 ^c^ ± 1.63	28.00 ^b^ ± 3.89	43.00 ^a^ ± 4.23
	Neuronal necrosis	0.00 ^c^ ± 0.00	4.00 ^c^ ± 1.63	10.00 ^b^ ± 2.11	23.00 ^a^ ± 3.00
Percentages of the area fractions of the lesion regarding the total areas of the images per group	Intracerebral blood vessels	2.30 ^d^ ± 0.23	3.44 ^c^ ± 0.21	4.96 ^b^ ± 0.34	6.47 ^a^ ± 0.29
	Meningeal blood vessels	0.70 ^d^ ± 0.09	1.19 ^c^ ± 0.11	1.79 ^b^ ± 0.15	2.82 ^a^ ± 0.12
	Choroidal plexus blood vessels	1.84 ^d^ ± 0.18	2.31 ^c^ ± 0.17	3.46 ^b^ ± 0.21	5.15 ^a^ ± 0.38
	Intracerebral hemorrhages	0.00 ^c^ ± 0.00	0.08 ^bc^± 0.04	0.54 ^ab^ ± 0.15	0.95 ^a^ ± 0.28
	Meningeal hemorrhages	0.00 ^c^ ± 0.00	0.38 ^bc^ ± 0.27	1.42 ^ab^ ± 0.53	1.70 ^a^ ± 0.49
	Neuropil microcavitations	0.00 ^b^ ± 0.00	0.69 ^b^ ± 0.18	4.10 ^a^ ± 0.58	5.82 ^a^ ± 1.06
The frequencies of the lesion regarding the total number of images per group	Glial clustering	0.00 ^b^ ± 0.00	9.00 ^a^ ± 3.14	4.00 ^ab^ ± 2.21	5.00 ^ab^ ± 2.24
	Gliosis	0.00 ^b^ ± 0.00	2.00 ^b^ ± 1.33	9.00 ^a^ ± 2.33	9.00 ^a^ ± 2.77
	Neuronophagia	0.00 ^c^ ± 0.00	1.11 ^bc^ ± 0.61	2.00 ^ab^ ± 0.71	3.00 ^a^ ± 0.73
	Endothelial hypertrophy	0.00 ^b^ ± 0.00	4.00 ^ab^ ± 1.63	7.00 ^a^ ± 2.60	8.00 ^a^ ± 2.00
	Cerebral inflammatory cell infiltration	0.00 ^b^ ± 0.00	1.00 ^ab^ ± 0.54	3.00 ^ab^ ± 1.27	4.00 ^a^ ± 1.63
	Meningeal inflammatory cell infiltration	0.00 ^b^ ± 0.00	0.80 ^ab^ ± 0.53	1.40 ^a^ ± 0.60	2.00 ^a^ ± 0.45

Values are represented as the mean ± SE. The means within the same row carrying different superscripts (^a–d^) are significant at *p* < 0.05.

## Data Availability

All datasets generated for this study are included in the article.
